# Targeted full energy and protein delivery in critically ill patients: a study protocol for a pilot randomised control trial (FEED Trial)

**DOI:** 10.1186/s40814-018-0249-9

**Published:** 2018-02-20

**Authors:** Kate Fetterplace, Adam M. Deane, Audrey Tierney, Lisa Beach, Laura D. Knight, Thomas Rechnitzer, Adrienne Forsyth, Marina Mourtzakis, Jeffrey Presneill, Christopher MacIsaac

**Affiliations:** 1 Department of Clinical Nutrition, Allied Health, Royal Melbourne Hospital, Melbourne, Australia; 2 Department of Rehabilitation, Nutrition and Sport, School of Allied Health, La Trobe University, Melbourne, Australia; 3 Department of Intensive Care Medicine, Royal Melbourne Hospital, Melbourne, Australia; 4 Department of Medicine, The University of Melbourne, Melbourne, Australia; 5 Department of Physiotherapy, Allied Health, Royal Melbourne Hospital, Melbourne, Australia; 6 Department of Kinesiology, Faculty of Applied Health Sciences, University of Waterloo, Waterloo, Canada

**Keywords:** Nutritional support, Enteral nutrition, Nutritional requirements, Dietary protein, Critical illness, Critical care, Intensive care

## Abstract

**Background:**

Current guidelines for the provision of protein for critically ill patients are based on incomplete evidence, due to limited data from randomised controlled trials. The present pilot randomised controlled trial is part of a program of work to expand knowledge about the clinical effects of protein delivery to critically ill patients. The primary aim of this pilot study is to determine whether an enteral feeding protocol using a volume target, with additional protein supplementation, delivers a greater amount of protein and energy to mechanically ventilated critically ill patients than a standard nutrition protocol. The secondary aims are to evaluate the potential effects of this feeding strategy on muscle mass and other patient-centred outcomes.

**Methods:**

This prospective, single-centred, pilot, randomised control trial will include 60 participants who are mechanically ventilated and can be enterally fed. Following informed consent, the participants receiving enteral nutrition in the intensive care unit (ICU) will be allocated using a randomisation algorithm in a 1:1 ratio to the intervention (high-protein daily volume-based feeding protocol, providing 25 kcal/kg and 1.5 g/kg protein) or standard care (hourly rate-based feeding protocol providing 25 kcal/kg and 1 g/kg protein). The co-primary outcomes are the average daily protein and energy delivered to the end of day 15 following randomisation. The secondary outcomes include change in quadriceps muscle layer thickness (QMLT) from baseline (prior to randomisation) to ICU discharge and other nutritional and patient-centred outcomes.

**Discussion:**

This trial aims to examine whether a volume-based feeding protocol with supplemental protein increases protein and energy delivery. The potential effect of such increases on muscle mass loss will be explored. These outcomes will assist in formulating larger randomised control trials to assess mortality and morbidity.

**Trial registration:**

Australian New Zealand Clinical Trials Registry (ANZCTR), ACTRN: 12615000876594 UTN: U1111-1172-8563.

## Background

Nutritional therapy, preferably via the enteral route, is part of the standard care for critically ill patients [1]. Prominent critical care nutrition guidelines recommend that protein should be provided at a level of 1.2–2.0 g/kg/day, with possibly higher amounts for patients with multi-trauma, obesity and burns and greater than 80% of energy targets should be met [1]; however, there is a lack of high-quality evidence to support these guidelines [2]. Despite these and similar guidelines, nutritional delivery in the intensive care unit (ICU) is frequently less than these targets; observational data from a large international dataset suggests that critically ill patients only receive, mean SD, 43 g ± 27 of protein and 1054 kcal ± 717 of energy per day, which equates to approximately 50 and 60% of their protein and energy targets, respectively [3]. More recent observational data from this dataset suggests that increasing protein delivery by 30 g, or meeting greater than 80% of prescribed protein targets, is associated with greater survival, an increase in ventilator-free days and a shorter time to discharge alive from the ICU [4, 5]. In addition, data from a prospective observational cohort study from a single centre suggested that the provision of more protein (greater than 1.5 g/kg/day) was associated with a reduction in mortality when adjusted for severity of illness and age [6]. Finally, observational study and preliminary trial data supporting the concept that increasing calorie delivery will improve outcomes [7–9].

Standard enteral feeding regimens are generally based on an hourly target rate of administration of a selected formulation, calculated according to daily energy and protein targets [10]. Therefore, if interruptions to feed delivery occur, protein and energy targets are not met [11]. Furthermore, protein delivery is generally restricted by the composition of the enteral formula available because overall energy requirements mostly determine the volume of the formula prescribed. In a cluster randomised control trial, Heyland and colleagues reported that with a novel approach to feeding, including a volume-based feeding protocol, delivery of protein increased by 14% (95% CI, 5–23%) and calories by 12% (95% CI, 5–20%) [11]. However, theoretically, volume-based feeding protocols with protein supplementation may not achieve greater delivery of protein and energy to patients due to issues with feeding intolerance, as increased nutrient delivery, particularly protein, to the small intestine, stimulates the feedback loop to slow gastric emptying [12, 13]. Therefore, this approach may inadvertently decrease protein and energy delivery.

Beyond mortality, patient-centred functional outcomes in survivors of critical illness are increasingly being recognised as important variables that may be influenced by nutrition [14, 15]. This includes muscle weakness, which is often described as Intensive Care Unit Acquired Weakness (ICUAW) [16]. Lower health-related quality of life (HRQoL) scores are associated with ICUAW, emphasising that weakness is important to patients [17–19]. The loss of skeletal muscle has been identified as a crucial contributing factor to the development of ICUAW [20, 21], with ultrasound being a potentially useful and minimally invasive modality to quantify muscle mass and muscle loss in the critically ill [22–24].

Augmenting nutrient delivery, particularly increased protein delivery, has been proposed as an approach that may attenuate muscle loss associated with critical illness. At present, there is an absence of robust data to support this approach [25] and recent studies have reported conflicting conclusions [6]. Most recently, Ferrie and colleagues [26] performed a randomised controlled trial in parenterally fed patients. Amongst other outcomes, they measured muscle mass and strength [26]. This study of 119 critically ill patients, who were randomised to receive a target of either 0.8 or 1.2 g/kg protein per day with isocaloric parenteral nutrition, did not find strong evidence of a difference in the primary outcome of handgrip strength at day 7, but the observed point estimate was in the direction of benefit for those receiving greater protein delivery. Moreover, the augmented protein intervention may have been associated with reduced fatigue and greater forearm muscle thickness using ultrasound [26]. A retrospective observational study of 106 critically ill patients, by Ishibashi and colleagues, reported that protein intake above 1.5 g/kg/day substantially reduced total body protein loss when compared to those who receive less than 1.1 g/kg/day [27]. In contrast, Casaer and colleagues reported that greater calorie and protein administration, particularly when delivered via the parenteral route, reduced the quantity and quality of muscle and was associated with greater muscle weakness [28]. This negative signal was also described by Puthucheary and colleagues, who reported greater muscle wasting associated with increased protein delivery [29].

At present, there is conflicting evidence around the optimal protein provision to critically ill patients including a lack of high-quality evidence which includes patient-centred functional outcomes. This single-centre pilot trial in mechanically ventilated critically ill patients is part of a program to explore the influence of protein prescription on outcomes; aims to determine whether a volume-based enteral feeding protocol with additional protein supplementation delivers a greater amount of protein and energy than a standard nutrition protocol.

## Study objectives

### Co-primary

The primary aim of this pilot study is to determine whether a volume-based feeding protocol with supplemental protein potentially improves the average daily protein and energy delivery, when compared to standard care in mechanically ventilated critically ill patients.

### Secondary

The secondary aims are to evaluate whether a volume-based feeding protocol with supplemental protein when compared to standard care:

I. Improves overall protein and energy adequacy without increasing feeding intolerance or diarrhoea.
II. Improves nutritional-related outcomes including the incidence of malnutrition at ICU discharge or mid upper arm circumference change from baseline to discharge.
III. Decreases the change in quadriceps muscle layer thickness (QMLT) from baseline to ICU discharge.
IV. Decreases the incidence of ICUAW or alters muscle strength or physical function scores at ICU discharge.
V. Alters the duration of the ICU admission, number of deaths, and the requirement for discharge to a rehabilitation facility.

## Methods/design

**Fig. 1:**
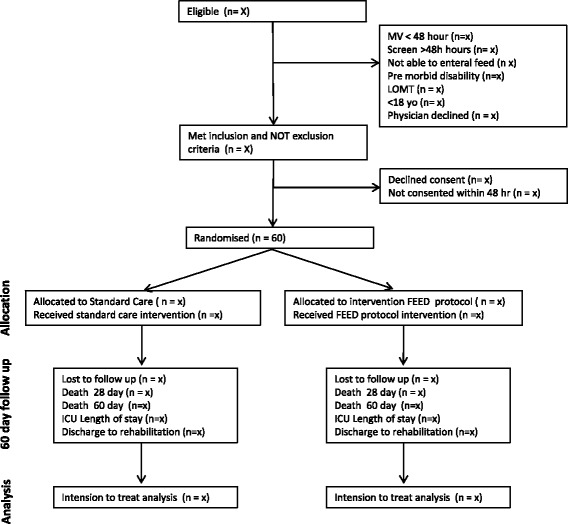
Study flow diagram: flow diagram of patients recruited and study conduct. Abbreviations: *MV* mechanically ventilated, *LOMT* limit of medical treatment, *yo* years old, *FEED Protocol* intervention

This pilot, single-centred, single-blinded, parallel group, prospective randomised controlled trial has been designed in accordance with the Standard Protocol Items: Recommendations for Interventional Trials (SPIRIT 2013) [30] and the Consolidated Standards for Reporting of Trials CONSORT guidelines [31] (Fig. 1, study flow diagram. The study will be undertaken at the Royal Melbourne Hospital ICU, which is a university-affiliated, tertiary referral, mixed medical-surgical-trauma ICU with 32-beds that has > 2500 patients admitted per year.

### Study participants

Sixty patients will be recruited within 48 h of their index ICU admission. Screening commenced on 10th of August 2015 and is performed only on weekdays. Patients who meet the study criteria (Table 1.) will be eligible to participate. As all eligible patients are mechanically ventilated and unable to consent to participation, informed consent will be obtained from the person responsible as per local laws. Consent to continue in the trial will be obtained from the participant if they recover adequately and they are deemed competent. The protocol and consent process has been approved by the Royal Melbourne Hospital Human Research Ethics Committee (2015.048). The protocol is registered with Australian New Zealand Clinical Trials Registry (ANZCTR; U1111-1172-85630).

**Table 1:** Inclusion and exclusion criteria

Inclusion	• Adults, ≥ 18 years of age • Mechanically ventilated (MV) for ≥ 48 h with no immediate plans to extubate in the next 24 h
Exclusion	• Patients who have a contraindication to enteral feeding • Limit of medical treatment order in place or imminent death • Pre-morbid disability causing inability to ambulate > 10 m independently (+/− gait aid) • Pregnancy • The treating clinician considers the intervention not in the patients best interest or too burdensome

### Baseline data collection

Baseline measurements will reflect the status of patients at or prior to randomisation. Demographic data includes admission diagnosis, comorbid illness including quantification using the Charlson Comorbidity Index [32], Katz Activities of Daily Living (ADL) index [33] (prior to the ICU admission), Acute Physiology and Chronic Health Evaluation II (APACHE II) score, and admission Sequential Organ Failure Assessment (SOFA) Score. Baseline measures, collected by the study Dietitian, include height (using ulna length [34]), weight (from bed scales), body mass index (BMI), mid upper arm circumference, nutritional status using the subjective global assessment (SGA) [35, 36], plasma albumin, highest and lowest blood glucose in the first 24 h of ICU admission, independent dietitian estimation of energy (weight-based or Schofield equation [37]) and protein requirements and the first quadriceps muscle layer thickness measure [24], see Table 3 for details of data collection.

### Randomisation and blinding

We will use a simple randomisation system to assign participants [1:1] to receive either standard care or the intervention. Allocation will be concealed using sequentially numbered opaque sealed envelopes, held by research personal not involved with the study. If the participant or the person responsible wishes to withdraw consent at any time, all study procedures will cease and the participant will receive standard ICU care as directed by the clinical team. Due to the nature of the intervention, the study is single-blinded. However, outcomes of muscle strength and physical function will be measured by an investigator blinded to the group allocation. Data analysis will be performed using a binary treatment code to maintain group allocation of blinding until the results are finalised.

### Trial intervention and comparator

The intervention or standard care will be delivered following randomisation until ICU discharge; the patient no longer requires enteral tube feeding, or at the end of day 15, with the day of randomisation being day 1. Tolerance of enteral nutrition will be assessed and managed similarly for both groups, with prokinetic drugs (metoclopramide 10 mg q.i.d. and erythromycin 200 mg b.d.) administered if gastric residue volumes at any time equal or exceed 300 ml [38]. The need for parenteral nutrition will be determined by treating clinical staff that are not investigators and are not aware of group allocations.

### Standard care group

The comparator group will receive standard nutrition care [39], which includes commencing a standard commercially available 1.0 kcal/ml enteral formula (Nutrison® 1.0 kcal, Nutricia, Wuxi, China), providing 40 g protein and 1000 kcal per litre. Liquid nutrient will be commenced using our ICU nutrition protocol (Appendix), and the target rate will be set at 25 kcal/kg ideal body weight (IBW) [1]. This strategy is designed to prescribe 1.0 g/kg protein and 25 kcal/kg of energy per day, however it is anticipated participants will receive less than this due to interruptions to nutrition therapy [39]. For participants below or within the IBW range (defined as a BMI between 18.5–25 kg/m^2^ for 18–65 years and 22–27 kg/m^2^ for ≥ 65 years [40]), actual body weight will be used. For participants with BMI ≥ 32 kg/m^2^, an adjusted IBW will be used (IBW + 25% (actual weight – IBW)) [41].

### Intervention group

**Fig. 2:**
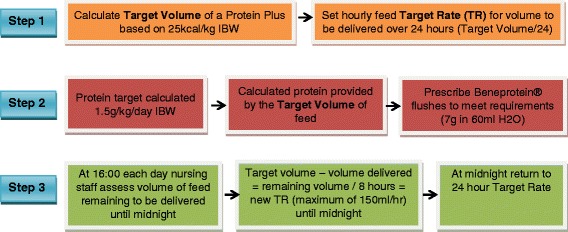
FEED protocol: how nutrition will be delivered in the intervention group. Abbreviations: *kg* kilogramme, *IBW* ideal body weight, *g* gram, *ml* millilitre, *H*_*2*_*0* water

The intervention group will receive their nutrition care based on the FEED protocol (Fig. 2, FEED protocol). It is anticipated that this will provide 1.5 g/kg protein and 25 kcal/kg of energy per day. The enteral formula will be a 1.25 kcal/ml formula (Nutrison® Protein plus, Nutricia, Zoetermeer, The Netherlands), providing 1250 kcal and 63 g protein per litre. The target formula volume will be determined based on 25 kcal/kg IBW, and the difference between the protein provided from the enteral formula and the target protein requirement of 1.5 g/kg IBW will be calculated. This difference will be met using protein powder (Beneprotein®, Nestle Health Sciences, Switzerland), provided in 6 g protein boluses (1 scoop of powder (7 g), provides 6 g of protein) over the day. Beneprotein® is a commercially available product that contains 100% whey protein isolate. To ensure that the target volume of formula is delivered, at 16:00 each day, the ICU bedside nurse will calculate the volume of formula provided, compared to the target volume, and then adjust the feed rate to aim to deliver the remaining feed volume over the 8-h period to midnight, with a maximum feeding rate of 150 ml/h [11]. The data on compliance to the feeding protocol will be collected.

### Management of fluid overload

For both groups, if the attending intensivist wishes to reduce the volume of feed provided, the feed will be changed to Nutrison® Concentrate 2.0 kcal/ml, (Nutricia, Zoetermeer, The Netherlands) with a goal to provide 25 kcal/kg of energy. For the standard care group, this will mean they will receive comparable protein delivery to the 1.0 kcal formula. For the intervention group, the protein supplementation will be increased in an attempt to achieve 1.5 g/kg/day. These changes will be recorded.

### Management of withdrawal

Stopping criteria are provided (Table 2), and any withdrawals will be recorded. The data will be retained and reported for all withdrawals where allowed by patient consent. Any adverse events will be recorded and reported.

**Table 2:** Criteria for withdrawal

Criteria	Measure
Feed intolerance	Tolerating < 40% of requirements via the enteral route for ≥ 3 days
Renal failure	If eGFR is < 25% and the patient is not commenced on continuous renal replacement therapy within 2 days
Severe oedema	> 5 L positive fluid balance, without alternative way to manage fluid balance and attending physician assesses the feed volume and additional protein to be impacting on the patients treatment after the above volume considerations have been implemented
Diarrhoea	≥ 500 ml per day or five bowel actions
Intensivist or attending physician request	No parameter
Participant/person responsible request to withdraw	No parameter

### Management of adverse events

It is not expected that any adverse events will occur in relation to the study protocol. However, each participant will be monitored regularly by study personnel for adverse events occurring throughout the study. If any adverse events do present the nature, severity, causality, and course of the adverse event will be recorded. Adverse events such as death, ischaemic bowel, renal failure, and diarrhoea will be recorded from the time of consent; if these events occur, this will be discussed with the attending ICU consultant to determine if they may be related to the study. Any severe adverse events related to the study will be reported to the Melbourne Health Human Research and Ethics Committee within 24 h of personnel becoming aware of it.

### Outcome measures

**Fig. 3:**
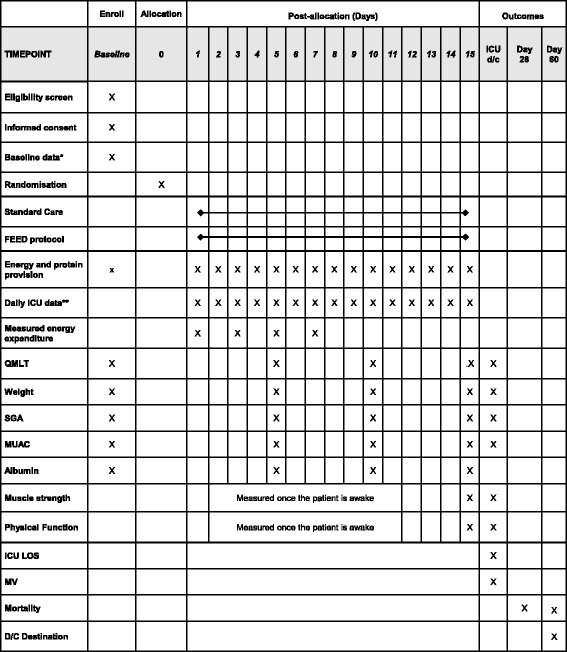
Study events, data collection, and outcome measures. *Baseline data includes age, gender, body mass index, admission diagnosis, Charlson Comorbidity Index, APACHE II score, SOFA Score, Katz Activities of Daily living (ADL) index, dietitian estimation of energy, and protein requirements. **Daily ICU data includes interruptions to feeding, fluid balance, feeding intolerance (gastric residual volumes), diarrhoea (> 300 ml per day), the presence of sepsis, renal failure, urea and creatinine levels, blood sugar levels and insulin dose (unit/day). Abbreviations: *FEED protocol* intervention, *ICU* intensive care unit, *QMLT* quadriceps muscle layer thickness, *SGA* subjective globe assessment, *MUAC* mid upper arm circumference, *LOS* length of stay, *MV* mechanical ventilation, *D/C* discharge

The data collection and the outcome measures are summarised in Fig. 3.

### Primary outcomes

The co-primary outcomes are mean daily protein and energy provided over the 15-day study period. The provision of protein and energy will be calculated on a daily basis and will be determined taking into account all sources; this includes nutrition therapy (enteral formula, protein supplements, and parenteral formula), dextrose, and propofol. The mean protein and energy provided will be determined, by adding the daily provision over the 15-day study period (the 14 complete calendar days). The provision of any nutrition prior to commencing the study protocol will be collected and reported but not included in the primary outcome analysis.

### Secondary outcomes

The first secondary outcome measurement is the change in muscle mass, determined as the change in QMLT using ultrasound from baseline to day 15 or ICU discharge if earlier. A portable ultrasound device (Sonosite S-ICU™) with a multiple frequency transducer (13-6 MHz, 6 cm) will be used to obtain muscle mass images. The method to obtain the images will be carried out as described by Tillquist and colleagues [24], with measurements completed using both minimal and maximal pressures. The first measure will be taken before randomisation, then on day 5, day 10, and at discharge or day 15 of ICU. A single-trained operator will complete all QMLT measures, and this technique has a very good intrarater reliability in both healthy populations [24] and critically ill patients [42]. The measurement will be completed on all participants, with bilateral measurements at two points; the midpoint between the Anterior Superior Iliac Spine (ASIS) and the upper pole of the patella and at the point two thirds between the ASIS and the top of the patella [22–24], the landmarks will be marked using a permanent pen. A linear measure will be taken for each point twice, with minimal pressure and maximal pressure applied and the measures will be recorded [22]. The device settings will be standardised for each measure, and bony landmarks will be used to determine if the transducer is oriented perpendicular to the muscle [24]. Each image will be stored to the hard drive of the ultrasound device and then transferred for further blinded analysis at a later date. The change in QMLT from baseline to day 5 and discharge or day 15 will be calculated for each patient for the measurements taken with minimal pressure and maximal pressure separately.

The other secondary outcomes are total calorie and protein provision, calorie deficit and protein adequacy, incidence of feed intolerance, number of days of feed intolerance, incidence of diarrhoea defined as more than three bowel actions or greater than 300 ml per day [43], length of ICU stay, hospital mortality at 28 and 60 days, discharge destination, muscle strength, the incidence of ICU AW and physical function at ICU discharge, and the change in nutritional markers over the study period. Nutritional markers include assessment of malnutrition using the Subjective Globe Assessment (SGA) [35], mid upper arm circumference (MUAC), and body weight. These measures will be carried out at baseline, day 5, and at discharge from ICU; the change from baseline to discharge or day 15 will be calculated. The presence of acute renal failure defined by the RIFLE criteria [44] and plasma urea and creatinine levels will be assessed on a daily basis.

Protein adequacy will be determined over the ICU admission or until day 15 by adding the daily protein provision and comparing this to the estimated protein requirements determined by the dietitian. The protein adequacy will then be explored in relation to the other outcome measures.

Calorie deficit will be determined using both predicted weight-based energy requirements (25 kcal/kg). In eligible patients, calorie deficit will also be calculated using measured energy expenditure (MEE) [45]. Cumulative calorie deficit will be calculated over the ICU admission or until day 15 of the study. MEE will be assessed with indirect calorimetry using E-sCOVX (GE, Helsinki, Finland) [46]. The first MEE will be within 24 h of enrolment into the study and repeated on day 3, day 5, and day 7 of the ICU stay. The measurements will be carried out with the patient in a fed state lying supine. The measure will be taken over a 2-h period, and the summary data of respiratory quotient (RQ) and MEE will be recorded. The feeding rates will not be adjusted in relation to the MEE [47]. Contraindications to carrying out a metabolic measurement will include if the patient is on continuous renal replacement therapy or extracorporeal membrane oxygenation (ECMO) or if the patient has an intercostal catheter with an air leak or is on a fraction of inspired oxygen greater than 0.6 [47].

Feed intolerance will be determined as a single gastric residual volume > 300 ml [48].

Muscle strength will be determined in suitable participants using handgrip dynamometry and the Medical Research Council (MRC) scale [49, 50]. The first muscle strength test will be performed at awakening [50] and then again at discharge or day 15, whichever comes first. Patients will be screened for attention and comprehension on the basis of their ability to follow commands, they will be considered awake if they score at least three out of five using the De jonghe comprehension criteria on at least two occasions within a 6-h period [51] and have a Riker sedation-agitation scale score of three to five [52]. Handgrip dynamometry (Commander Echo™ Wireless Grip Dynamometer, USA) will be measured in both limbs with the participant in a chair or sitting at least at 45° in bed, with the patients elbow at 90° supported by a pillow or the arm of the chair. The Medical Research Council sum score (MRC-SS) will be measured as previously described [51, 53, 54] with ICUAW defined as an MRC-SS of < 48/60 [55].

Physical function will also be assessed using the physical function in Intensive Care Test–scored (PFIT-s) [56, 57]. Patients may only be able to perform part of the test, but are still able to obtain a score.

### Sample size

A sample size of 60 is based on observational data [45], where daily protein intake, mean (SD), was 50.8 g (20.1 g) protein per day; therefore, 29 participants per group will provide 80% power (two-sided *α* 0.05) to detect a minimum difference of a 15-g protein between groups. While there is limited data on change in muscle mass using ultrasound, using the data from the VALIDUM study, mean (SD) QMLT of 1.3 (0.6) cm [22], a sample size of 28 participants in each group will also provide over 80% power to detect a mean difference of 0.5 cm in QMLT.

### Statistical analysis

All analyses will use an intention-to-treat approach. Baseline patient demographics, severity of illness, ICU length of stay, mortality, and nutritional markers will be tabulated according to the treatment group. Initial exploratory data analysis will involve calculation of summary statistics and comparison between treatment groups using non-parametric (Wilcoxon), parametric (*t* test), and Fisher’s exact tests as appropriate, as well as construction of trajectory plots according to the treatment group. The co-primary outcomes of average daily energy and protein delivery will be compared between treatment and control groups with two-sample unpaired *t* tests, with statistical significance for each set conservatively at two-sided values of 0.025 to limit the family-wise type 1 error for the two co-primary outcomes to less than 0.05 overall.

All secondary outcomes will be regarded as exploratory and hypothesis-generating, with no multiple comparison adjustment to conventional 5% type 1 error thresholds. Group differences for change from baseline at selected time points in continuous outcome variables, including QMLT, will be compared after adjustment for initial values using analysis of covariance (ANCOVA) regression models, initially unadjusted, and subsequently adjusted as described below for other regression models. Natural logarithmic transformations may be applied to stabilise variance within these or other linear models if appropriate. The relationships between calorie deficits using both prescribed calories (25 kcal/kg) and MEE and protein adequacy and the outcome measures (QMLT change, muscle strength, diagnosis of ICUAW, and physical function at ICU discharge) will be explored using linear or logistic regression analyses, with adjustment for likely confounders and any baseline variable found to show substantial imbalance between treatment groups. Finally, a population-averaged generalised linear model using a generalised estimating equation (GEE) approach with an unstructured working correlation matrix and robust standard error estimates adjusted for clustering within individual subjects will be applied to these longitudinal data to evaluate the overall associations of the vector of (untransformed or log transformed) outcome variables with treatment group across multiple time points. Multiple imputations for missing data may be used to support conclusions from other generalised linear models constructed without imputation of missing data. Data analysis will be carried out using the Statistical Package for the Social Sciences (SPSS) (IBM® SPSS® Statistics Premium Grad Pack Version 22.0) or Stata Corporation (Stata Statistical Software: Release 15. College Station, TX: StataCorp LP; 2017).

## Discussion

It has been hypothesised that protein provision may be more important than energy provision for critically ill patients [25]. Optimal protein delivery may also influence functional outcomes via attenuation of muscle loss. However, the optimal amount of enteral protein required in critical illness is relatively unknown and has not been rigorously studied. Moreover, attempts to augment enteral protein delivery may result in slower gastric emptying, greater feeding intolerance, and, thereby, paradoxically less protein and calorie delivery.

The strengths of this study include randomisation, comparing two different protein amounts using enteral nutrition, and assessment of the impact of nutrition provision on muscle mass and functional outcomes that is essential for planning a larger multicentre study.

This pilot study aims to clarify whether an enteral feeding protocol with a volume target and supplemental protein has potential to increase protein and energy delivery in mechanically ventilated critically ill patients and will provide preliminary estimates as to whether this intervention has the capacity to affect muscle mass or other patient-centred outcomes.

## Figures and Tables

**Fig. 4:**
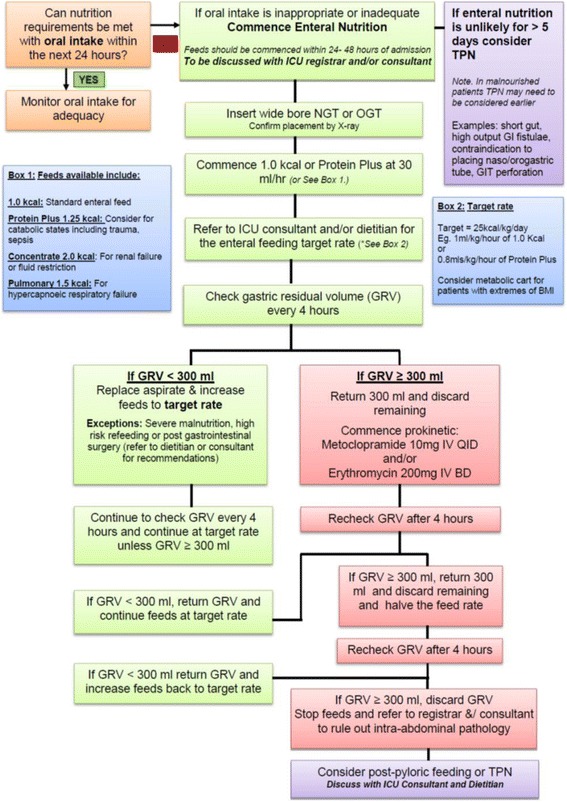
RMH ICU enteral feeding procedure

## Abbreviations

ADL: Activities of daily living
APACHE: Acute Physiology and Chronic Health Evaluation
BMI: Body mass index
CONSORT: Consolidated Standards for Reporting of Trials
ECMO: Extracorporeal membrane oxygenation
GEE: Generalized estimating equation
HRQoL: Health-related quality of life
IBW: Ideal body weight
ICU: Intensive care unit
ICUAW: Intensive care unit acquired weakness
MEE: Measured using energy expenditure
MRC: Medical Research Council
MRC-SS: Medical Research Council sum score
MUAC: Mid Upper Arm Circumference
MV: Mechanically ventilated
PFET-s: Physical Function in Intensive Care Test – scored
QMLT: Quadriceps muscle layer thickness
RQ: Respiratory quotient
SD: Standard deviation
SGA: Subjective global assessment
SOFA: Sequential Organ Failure Assessment
